# Nuclear membrane-localised NOX4D generates pro-survival ROS in FLT3-ITD-expressing AML

**DOI:** 10.18632/oncotarget.22241

**Published:** 2017-11-01

**Authors:** Jennifer N. Moloney, Ashok Kumar Jayavelu, Joanna Stanicka, Sarah L. Roche, Rebecca L. O'Brien, Sebastian Scholl, Frank-D. Böhmer, Thomas G. Cotter

**Affiliations:** ^1^ Tumour Biology Laboratory, School of Biochemistry and Cell Biology, Bioscience Research Institute, University College Cork, Cork, Ireland; ^2^ Institute of Molecular Cell Biology, CMB, Jena University Hospital, Jena, Germany; ^3^ Current address: Department of Proteomics and Signal Transduction, Max-Planck Institute of Biochemistry, Martinsried, Germany; ^4^ Department of Haematology/Oncology, Clinic for Internal Medicine II, Jena University Hospital, Jena, Germany

**Keywords:** acute myeloid leukaemia, FLT3-ITD, pro-survival reactive oxygen species, NOX4 splice variant D/NOX4D 28 kDa, nuclear membrane

## Abstract

Internal tandem duplication of the juxtamembrane domain of FMS-like tyrosine kinase 3 (FLT3-ITD) is the most prevalent genetic aberration present in 20-30% of acute myeloid leukaemia (AML) cases and is associated with a poor prognosis. FLT3-ITD expressing cells express elevated levels of NADPH oxidase 4 (NOX4)-generated pro-survival hydrogen peroxide (H_2_O_2_) contributing to increased levels of DNA oxidation and double strand breaks. NOX4 is constitutively active and has been found to have various isoforms expressed at multiple locations within a cell. The purpose of this study was to investigate the expression, localisation and regulation of NOX4 28 kDa splice variant, NOX4D. NOX4D has previously been shown to localise to the nucleus and nucleolus in various cell types and is implicated in the generation of reactive oxygen species (ROS) and DNA damage. Here, we demonstrate that FLT3-ITD expressing-AML patient samples as well as -cell lines express the NOX4D isoform resulting in elevated H_2_O_2_ levels compared to FLT3-WT expressing cells, as quantified by flow cytometry. Cell fractionation indicated that NOX4D is nuclear membrane-localised in FLT3-ITD expressing cells. Treatment of MV4-11 cells with receptor trafficking inhibitors, tunicamycin and brefeldin A, resulted in deglycosylation of NOX4 and NOX4D. Inhibition of the FLT3 receptor revealed that the FLT3-ITD oncogene is responsible for the production of NOX4D-generated H_2_O_2_ in AML. We found that inhibition of the PI3K/AKT and STAT5 pathways resulted in down-regulation of NOX4D-generated pro-survival ROS. Taken together these findings indicate that nuclear membrane-localised NOX4D-generated pro-survival H_2_O_2_ may be contributing to genetic instability in FLT3-ITD expressing AML.

## INTRODUCTION

Aberrant signalling of receptor tyrosine kinases (RTKs) is associated with tumour development and transformation [1–3]. FMS-like tyrosine kinase 3 (FLT3) is a type III RTK expressed in approximately 90% of acute myeloid leukaemia (AML) and plays a critical role in normal haematopoiesis [4–6]. Internal tandem duplication (ITD) of sequences in the juxtamembrane domain is the most prevalent genetic aberration of FLT3, with a gain of function mutation, implicated in 20-30% of AML patients [5, 7–10]. Patients with this mutation have a particularly poor prognosis with a high incidence of relapse [11–13]. Constitutive activation of the tyrosine kinase domain of FLT3-ITD results in autophosphorylation and activation of downstream pro-survival cascades including PI3K/AKT, ERK1/2 and STAT5 resulting in the promotion of cell survival, proliferation and transformation in myeloid leukaemia [9, 10, 14–19]. It has been demonstrated that AML cells expressing the FLT3-ITD mutation produce higher levels of reactive oxygen species (ROS) and DNA damage compared to their wild-type counterpart [20–23].

ROS have been long implicated in leukaemia cancer pathology due to their ability to induce DNA damage [24, 25]. The NADPH oxidase (NOX) family consisting of NOX1-5 and dual oxidase (DUOX) 1 and 2, are well established producers of ROS [26], with NOX2 and NOX4 playing a central role in the increased production of hydrogen peroxide (H_2_O_2_) in AML [23, 27, 28]. NOX proteins vary in structure, subcellular localisation, biochemical characteristics and regulatory subunits (p22^phox^, p47^phox^, p67^phox^ and Rac1/2). p22 phagocyte oxidase (p22^phox^) is a partner protein and is required for functionally active NOX1-4 [29–31]. Among the NOX family members NOX4 is unique. It is constitutively activated, generating H_2_O_2_, unlike its family members NOX1 and NOX2, which require an agonist for activation [32–34]. Oxidation of protein tyrosine phosphatases (PTPs) occurs in FLT3-ITD expressing AML cells. NOX4-driven ROS formation causes partial inactivation of DEP-1/PTPRJ, a transmembrane PTP responsible for negative regulation of FLT3 signalling, contributing to unfavourable downstream signalling [27].

NOX4 subcellular localisation plays an important role, given its constitutive activity. NOX4 has been reported to be expressed in the cytoskeleton [35], endoplasmic reticulum (ER) [30, 36–38], mitochondria [39, 40], plasma membrane [38, 41] and nucleus [42–44] in different cell types. Previous studies in our laboratory have shown that NOX4 and p22^phox^ co-localise to the nuclear membrane by immunofluorescence in FLT3-ITD expressing MV4-11 AML cell line contributing to DNA oxidation and double strand breaks (dsbs), possibly driving genetic instability [23].

Previous studies identified NOX4 isoforms, expressed at varying levels, in the presence of the prototype in the human lung cancer cell line, A549 cells. The truncated NOX4 splice variant D (28 kDa) lacks the majority of the transmembrane domain and has been shown to produce higher levels of ROS and DNA damage compared to its prototype [45]. NOX4D retains the NADPH and FAD-binding domains required for electron transfer activity and ROS production despite its truncation [46]. NOX4D is localised to the nucleus and nucleolus in vascular smooth muscle cells (VSMC), A7R5 cells and in many other cells including human aortic vascular smooth muscle cells, human umbilical vein endothelium cells (HUVEC), H9C2 rat cardiomyocytes, human embryonic kidney fibroblasts (HEK), mouse primary cardiac fibroblasts and rat neonatal cardiomyocytes. NOX4D is expected to be soluble rather than membrane-localised [42].

To investigate if FLT3-ITD expressing AML cells express NOX4D and in order to identify the localisation of NOX4D we utilised subcellular fractionation, inhibitors of FLT3-ITD and pro-survival signalling pathways, siRNA, alongside ROS specific probes and antibodies. Experiments were carried out in *de novo* primary AML samples, human patient-derived AML cell line MV4-11 and in the murine haematopoietic 32D cell lines stably harbouring FLT3-wild type (FLT3-WT) receptor and FLT3-ITD mutation.

We show that FLT3-ITD expressing AML patient samples and cell lines express the NOX4D 28 kDa splice variant. FLT3-ITD expressing AML cells express NOX4D in the nuclear membrane, which may be contributing to genetic instability in AML. NOX4D expression is dependent on the FLT3-ITD mutation. NOX4 partner protein p22^phox^ does not regulate NOX4 or NOX4D protein expression. Inhibition of the PI3K and STAT5 pro-survival pathways results in decreased expression of NOX4D alongside a decrease in endogenous H_2_O_2_ detected using the H_2_O_2_ specific probe Peroxy Orange 1 (PO1). Inhibition of ERK1/2 signalling had no effect on NOX4D protein expression, however a decrease in p22^phox^ protein levels alongside a decrease in endogenous H_2_O_2_ was observed. Inhibition of GSK3β resulted in increased expression of NOX4 and NOX4D, however, a slight decrease in endogenous H_2_O_2_ was observed. This demonstrates that NOX4D is downstream of FLT3-ITD signalling in AML, located in the nuclear membrane where it may be contributing to DNA damage and disease progression.

## RESULTS

### FLT3-ITD expressing AML patient samples, MV4-11 and 32D/FLT3-ITD cells express the NOX4 splice variant NOX4D 28 kDa in the nuclear membrane

**Figure 1:**
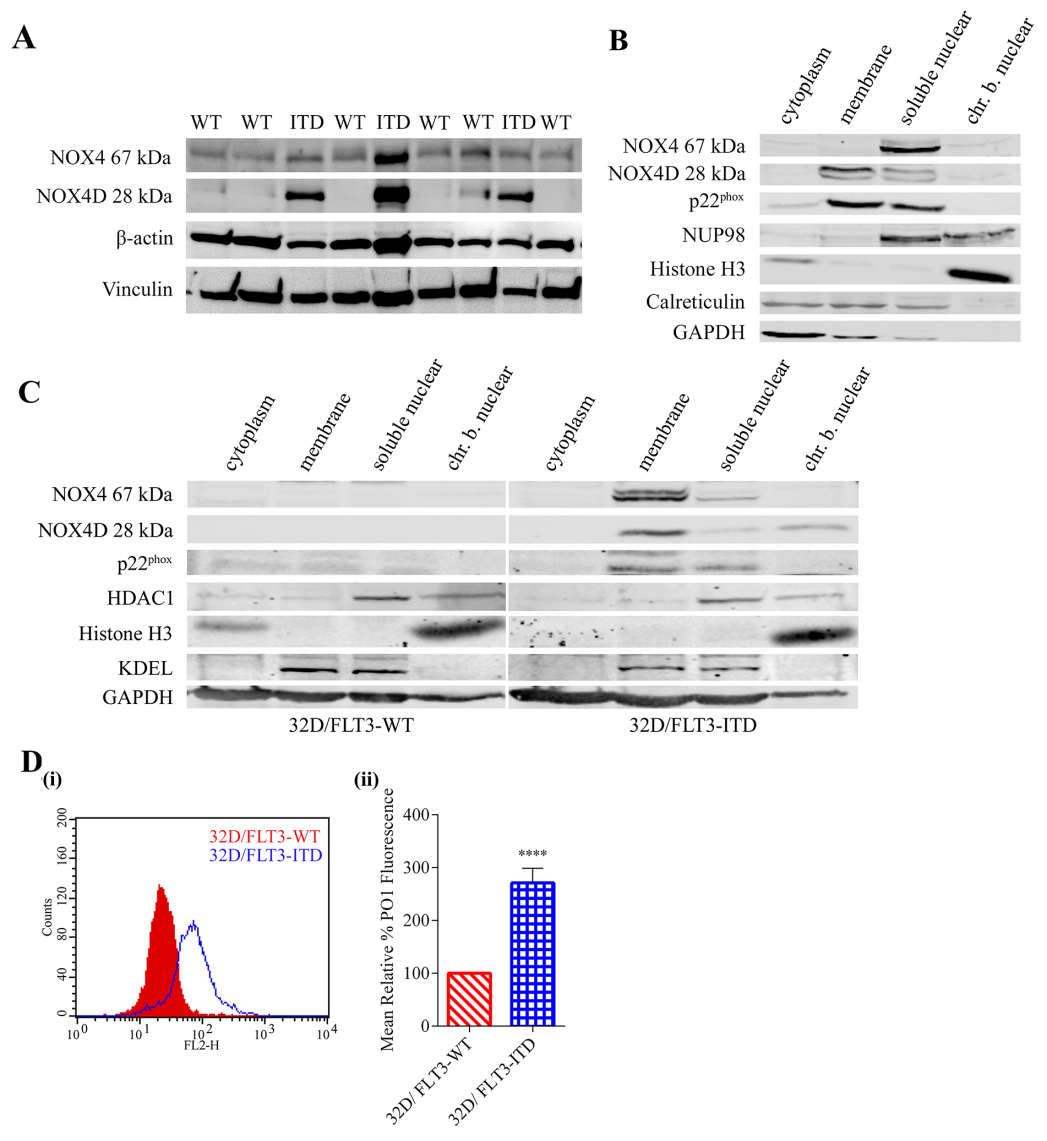
FLT3-ITD expressing AML patient samples and cell lines express the NOX4D 28 kDa isoform **(A)** Western blot analysis of NOX4 67 kDa and NOX4D 28 kDa protein expression in FLT3-ITD- and FLT-WT-expressing AML patient samples. β-actin and Vinculin were used as loading controls. **(B and C)** Subcellular fractionation was carried out in FLT3-ITD expressing AML cell line, MV4-11 **(B)** and 32D cells transfected with FLT3-WT or FLT3-ITD **(C)**. Expression of NOX4 67 kDa, NOX4D 28 kDa and p22^phox^ was assessed by means of western blot analysis. Equal loading of samples and verification of the subcellular fractions were demonstrated by probing for nuclear-localised NUP98, HDAC1 and Histone H3, membrane-localised calreticulin and KDEL and cytosolic-localised GAPDH. Blots are representative of five independent experiments. **(D) (i)** 32D cells stably transfected with FLT3-WT and FLT3-ITD were IL-3 starved overnight, followed by ROS visualisation with H_2_O_2_ specific probe, Peroxy Orange 1 (PO1) for 1 h before flow cytometric analysis. **(ii)** Bar chart shows relative mean PO1 fluorescence of 32D/FLT3-ITD cells expressed as a % of 32D/FLT3-WT cells. The results are representative of three independent experiments. Asterisks indicate statistically significant differences (^****^p<0.0001) as analysed by Student's t-test. Error bars represent SD.

FLT3-ITD expressing AML cells have been shown previously to express higher levels of total endogenous H_2_O_2_, DNA oxidation and dsbs compared to FLT3-WT cells [8, 23]. NOX4 has been well established as a producer of pro-survival ROS in FLT3-ITD expressing AML, contributing to DNA damage and disease progression [23, 27]. As mentioned previously, NOX4 is unique to other members of the NOX family of proteins in its constitutive activation. Therefore, NOX4 subcellular localisation plays an important role in cellular regulation. Our group has previously shown that NOX4 and p22^phox^ co-localise to the nuclear membrane in MV4-11 cells [23]. Previous studies identified the presence of NOX4 isoforms, including NOX4 splice variant NOX4D (28 kDa), to be expressed and localised to the nucleus and nucleolus of VSMC where it is contributing to ROS production, DNA damage and genetic instability [42]. We investigated if FLT3-ITD- and FLT3-WT-expressing AML patient samples expressed the NOX4D isoform and also examined the expression and localisation of NOX4D 28 kDa in two cell lines: FLT3-ITD-expressing AML MV4-11 cell line and 32D cell line stably transfected with FLT3-WT or FLT3-ITD. Localisation of NOX4D was assessed by means of subcellular fractionation. We show that NOX4D is expressed in FLT3-ITD expressing patient samples and cells, but is absent in FLT3-WT patient samples and 32D cells transfected with the FLT3-WT receptor (Figure 1A-1C). NOX4D is localised to the membrane and soluble nuclear fractions of MV4-11 cells (Figure 1B) and the membrane, soluble nuclear and chromatin bound nuclear (chr.b.nuclear) fractions of 32D cells stably transfected with FLT3-ITD (Figure 1C). In support of previous work, we have identified the NOX4 prototype (67 kDa) in the soluble nuclear fraction and p22^phox^ in the membrane and soluble nuclear fractions in both MV4-11 cells (Figure 1B) and 32D/FLT3-ITD cells (Figure 1C). Interestingly, we found NOX4 67 kDa was lacking from the membrane fraction in MV4-11 cells (Figure 1B). In contrast NOX4 67 kDa was observed in the membrane fraction of 32D/FLT3-ITD cells (Figure 1C). There are therefore clear differences in NOX4 67 kDa subcellular localisation between these cell lines.

### Specificity of NOX4 antibodies

Studies have raised issues with NOX4 antibody specificity in the past, due to difficulties in detecting NOX4 protein expression. Therefore, a key issue concerned the use of the Abcam NOX4 antibody (Ab109225) and the Novus Biologicals NOX4 antibody (NB110-58849) in our study. For this reason, a series of control experiments were first performed to validate the NOX4 antibodies employed in this study.

To validate the specificity of the Abcam NOX4 antibody (Ab109225) against NOX4 67 kDa and NOX4D 28 kDa in primary AML samples in this study, NOX4 knockdowns were carried out in 32D/FLT3-ITD cells. Specific NOX4 knockdowns using NOX4 targeted siRNA and shRNA in 32D/FLT3-ITD cells resulted in depletion of NOX4 67 kDa protein levels detected using Abcam NOX4 antibody (Supplementary Figure 1A). The remainder of the experiments in this study employed the Novus Biologicals NOX4 antibody (NB110-58849). In order to demonstrate specificity of this antibody for NOX4 67 kDa and NOX4D 28 kDa, HEK-293-T cells were transfected with empty vector (EV)-HA or p-CMV3-C-HA encoding NOX4 and were analysed 48 h post transfection by western blot. NOX4 overexpression in HEK 293-T cells resulted in increased NOX4 67 kDa and NOX4D 28 kDa protein expression in the presence of HA protein expression at the corresponding molecular weights compared to HEK 293-T cells transfected with EV (Supplementary Figure 1B). Together, these experiments validated that the NOX4 antibodies used in this study were specific for NOX4 67 kDa and NOX4D 28 kDa.

### 32D cells stably transfected with FLT3-ITD express higher levels of endogenous H_2_O_2_ compared to FLT3-WT

FLT3-ITD expressing AML cells have been shown to produce elevated levels of ROS and DNA damage compared to their wild type counterpart [23]. Having confirmed that FLT3-ITD expressing AML patient samples and 32D cells express NOX4D whereas FLT3-WT expressing patient samples and 32D cells do not, we investigated the effect of NOX4D expression on total endogenous H_2_O_2_. We demonstrate that 32D cells stably transfected with FLT3-ITD produced ∼170% more endogenous H_2_O_2_ than 32D cells stably transfected with FLT3-WT receptor, as assessed with a H_2_O_2_ specific probe-PO1 (Figure 1D).

### p22^phox^ knockdown had no effect on NOX4 67 kDa and NOX4D 28 kDa protein levels

**Figure 2:**
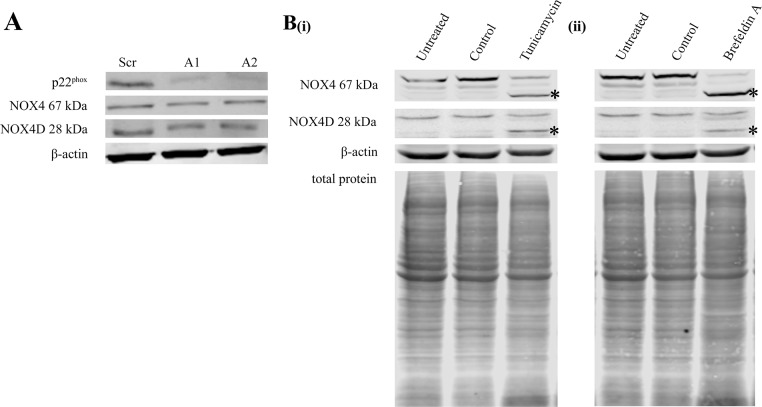
Knockdown of p22^phox^ in FLT3-ITD expressing MV4-11 cells had no effect on NOX4 67 kDa and NOX4D 28 kDa protein expression. NOX4D 28 kDa is glycosylated in FLT3-ITD expressing AML **(A)** Western blot analysis of p22^phox^, NOX4 67 kDa and NOX4D 28 kDa protein levels in MV4-11 whole cell lysates at 24 h following p22^phox^ siRNA transfection. β-actin was used as a loading control. **(B)** Western blot analysis of NOX4 67 kDa and NOX4D 28 kDa protein expression in whole cell lysates following treatment with tunicamycin (5 μg/ml) **(i)** and brefeldin A (10 μg/ml) **(ii)** overnight. (Asterisks indicate a shift in protein molecular weight). β-actin and total protein were used as loading controls. Blots are representative of three independent experiments.

p22^phox^ is a partner protein and is required for functionally active NOX1-4 [30]. Specific p22^phox^ knockdown allowed us to investigate the effects of p22^phox^ on NOX4 67 kDa and NOX4D 28 kDa protein expression. Knockdown of p22^phox^ had no effect on NOX4 67 kDa and NOX4D 28 kDa protein levels (Figure 2A).

### Inhibition of glycosylation in MV4-11 cell line resulted in NOX4 67 kDa and NOX4D 28 kDa deglycosylation

Previous studies in our laboratory have found NOX4 67 kDa to be glycosylated in FLT3-ITD expressing AML MV4-11 cells [28]. We investigated if NOX4D 28 kDa is glycosylated in the MV4-11 cell line. Cells were treated with glycosylation inhibitor, tunicamycin and receptor trafficking inhibitor, brefeldin A. Treatment of MV4-11 cells with glycosylation and receptor trafficking inhibitors resulted in deglycosylation of NOX4D 28 kDa as seen by the presence of a lower molecular weight band marked by asterisks (Figure 2B).

### Inhibition of FLT3-ITD in MV4-11 cell line and 32D cells transfected with FLT3-ITD causes a decrease in NOX4 67 kDa and NOX4D 28 kDa protein levels as well as reductions in total endogenous H_2_O_2_

**Figure 3:**
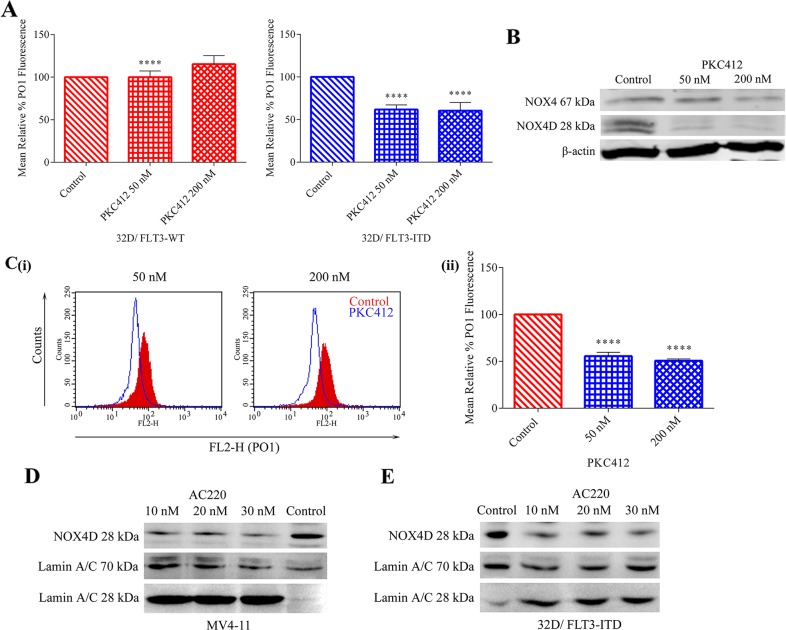
Inhibition of the FLT3 receptor in 32D/FLT3-ITD cells and FLT3-ITD expressing MV4-11 cells reduces NOX4 67 kDa and NOX4D 28 kDa protein expression and total endogenous H_2_O_2_ Inhibition of the FLT3 receptor following treatment with PKC412, results in a decrease in total endogenous H_2_O_2_ in 32D/FLT3-ITD cells but not in 32D/FLT3-WT cells. **(A)** 32D/FLT3-WT and 32D/FLT3-ITD cells were IL-3 starved overnight and treated for 24 h with PKC412 (50 nM and 200 nM), followed by staining with H_2_O_2_ specific probe PO1 for 1 h before FACS reading. Bar charts show relative mean PO1 fluorescence of treated cells expressed as % of control. The results are representative of four independent experiments. Asterisks indicate statistically significant differences (^****^p<0.0001) as analysed by Student's t-test. Error bars represent SD. **(B)** Western blot analysis of NOX4 67 kDa and NOX4D 28 kDa protein expression in MV4-11 whole cell lysates following treatment with PKC412 for 24 h at indicated concentrations. β-actin was used as a loading control. Blots are representative of three independent experiments. **(C) (i)** Flow cytometric analysis of mean relative PO1 fluorescence in MV4-11 cells treated with PKC412 for 24 h at indicated concentrations. **(ii)** Bar chart shows relative mean PO1 fluorescence of treated cells expressed as % of control. Results are representative of three independent experiments. Asterisks indicate statistically significant differences (^****^p<0.0001) as analysed by Student's t-test. Error bars represent SD. **(D and E)** Western blot analysis of NOX4D 28 kDa protein expression in the nuclear fraction following treatment with AC220 (10 nM, 20 nM and 30 nM) for 12 h in MV4-11 cells (D) and 32D cells transfected with FLT3-ITD (E). Equal loading of nuclear fractions was demonstrated by probing for nuclear-localised Lamin A/C. Blots are representative of three independent experiments.

32D cells transfected with FLT3-ITD and FLT3-WT receptor along with MV4-11 cells were treated with FLT3-ITD inhibitor, PKC412. The inhibition of FLT3 receptor resulted in a decrease in total endogenous H_2_O_2_ in 32D/FLT3-ITD cells, but not in 32D/FLT3-WT cells. As shown previously in Figure 1D 32D/FLT3-ITD cells possess ∼170% more total endogenous H_2_O_2_ compared to their wild-type counterpart. Moreover, inhibition of FLT3 resulted in ∼40% decrease in total endogenous H_2_O_2_ following treatment with 50 nM and 200 nM PKC412 specifically in 32D cells expressing the FLT3-ITD mutation and not in cells expressing FLT3-WT receptor (Figure 3A). FLT3-ITD expressing MV4-11 cells were treated with PKC412. Inhibition of the FLT3 receptor in MV4-11 cells resulted in decreased NOX4 67 kDa and NOX4D 28 kDa protein levels in whole cell lysates (Figure 3B) and reduced total endogenous H_2_O_2_. 50 nM and 200 nM PKC412 treatments resulted in a decrease of 45-50% in total endogenous H_2_O_2_ (Figure 3C). The inhibition of FLT3-ITD using AC220, another commonly used and very selective FLT3 receptor inhibitor, resulted in decreased NOX4D 28 kDa protein levels in the nuclear fractions of MV4-11 cells (Figure 3D ) and 32D/FLT3-ITD cells (Figure 3E). In Figure 3D and 3E Lamin A/C is used as a marker for nuclear fractions. Lamin A/C is cleaved by caspase-6 and serves as a marker of caspase-6 activation. During apoptosis, Lamin A/C is specifically cleaved to a large (40-45 kDa) and a small (28 kDa) fragment. The cleavage of Lamin A/C results in nuclear dysregulation and death. These results suggest that both FLT3-ITD and NOX4D proteins play a role in the generation of H_2_O_2_ in MV4-11 and 32D/FLT3-ITD cells and that FLT3-ITD activity is presumably an upstream regulator of NOX4D-generated pro-survival ROS.

### PI3K/AKT pathway is required for FLT3-ITD mediated-NOX4 67 kDa and -NOX4D 28 kDa generation of pro-survival H_2_O_2_

**Figure 4:**
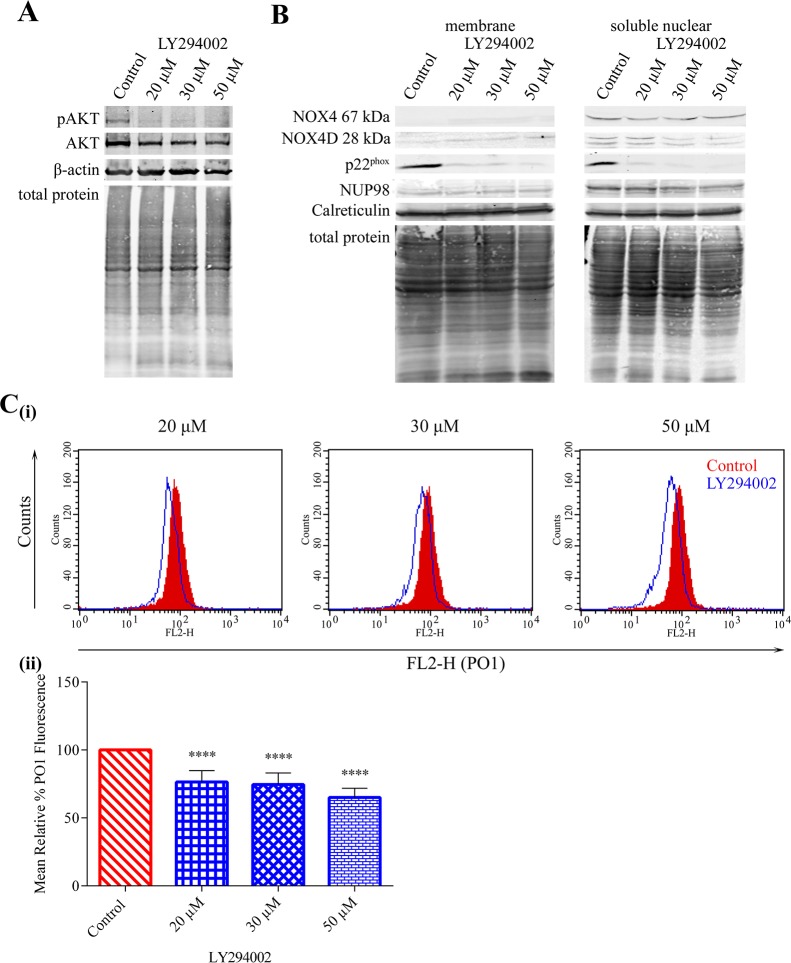
NOX4 67 kDa- and NOX4D 28 kDa–generated pro-survival ROS require AKT activation **(A)** Western blot analysis of AKT signalling in FLT3-ITD expressing MV4-11 cells following treatment with LY294002 (20 μM, 30 μM and 50 μM) for 16 h. β-actin and total protein were used as loading controls. **(B)** NOX4 67 kDa, NOX4D 28 kDa and p22^phox^ protein expression in membrane and soluble nuclear fractions of MV4-11 cells following treatment with LY294002 for 16 h at indicated concentrations. Equal loading of samples is shown by total protein and verification of subcellular fractions were assessed by probing for nuclear-localised NUP98 and membrane-localised calreticulin. Western blot analysis is representative of three independent experiments. **(C) (i)** Flow cytometric analysis of mean relative PO1 fluorescence in MV4-11 cells treated with LY294002 for 16 h at indicated concentrations. **(ii)** Bar chart shows relative mean PO1 fluorescence of treated cells expressed as % of control. Results are representative of three independent experiments. Asterisks indicate statistically significant differences (^****^p<0.0001) as analysed by Student's t-test. Error bars are representative of SD.

Constitutively activated FLT3-ITD kinase stimulates aberrant proliferative signalling through downstream signalling pathways including PI3K/AKT, ERK1/2, STAT5 and GSK3β. We have shown previously that NOX4- and p22^phox^-generated pro-survival ROS require AKT activation in MV4-11 cells [28]. Therefore, we examined the effect of PI3K/AKT inhibition on NOX4D 28 kDa protein expression in membrane and soluble nuclear fractions of MV4-11 cells. Inhibition of AKT (Figure 4A) using PI3K inhibitor LY294002 resulted in slight decreases in NOX4 67 kDa protein expression and more noticeable decreases in NOX4D 28 kDa and p22^phox^ protein expression in the soluble nuclear fraction (Figure 4B). As shown, NOX4D 28 kDa protein expression is weak in the membrane fraction and it is therefore difficult to detect any change. We next examined the effect of AKT inhibition on the generation of total endogenous H_2_O_2_. AKT inhibition resulted in ∼25% decrease in total endogenous H_2_O_2_ following treatment with 20 μM and 30 μM LY294002 and ∼35% decrease following treatment with 50 μM LY294002 (Figure 4C). Thus, activation of the AKT pathway is required for FLT3-ITD to produce NOX4D-generated ROS.

### NOX4 67 kDa- and NOX4D 28 kDa-generated pro-survival ROS are independent of ERK1/2 signalling however p22^phox^-mediated H_2_O_2_ production requires ERK1/2 activation

**Figure 5:**
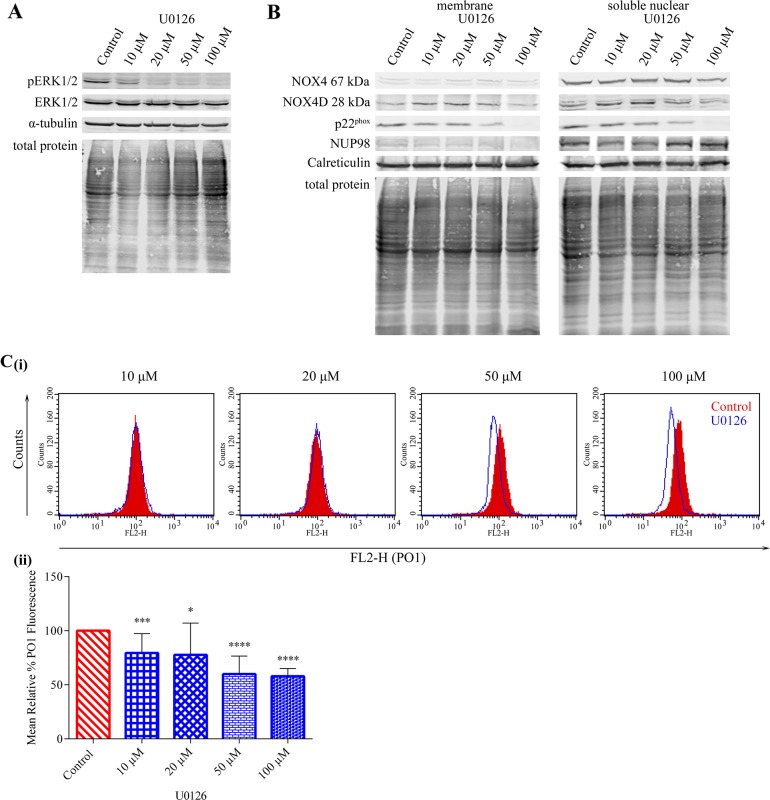
NOX4 67 kDa- and NOX4D 28 kDa-generated pro-survival ROS are independent of ERK1/2 signalling. p22^phox^ generated H_2_O_2_ requires ERK1/2 activation **(A)** Western blot analysis of ERK1/2 signalling in FLT3-ITD expressing MV4-11 cells following treatment with U0126 (10 μM, 20 μM, 50 μM and 100 μM) for 16 h. α-tubulin and total protein were used as loading controls. **(B)** NOX4 67 kDa, NOX4D 28 kDa and p22^phox^ protein expression in membrane and soluble nuclear fractions of MV4-11 cells following treatment with U0126 for 16 h at indicated concentrations. Equal loading of samples is shown by total protein and verification of subcellular fractions was assessed by probing for nuclear-localised NUP98 and membrane-localised calreticulin. Western blot analysis is representative of three independent experiments. **(C) (i)** Flow cytometric analysis of mean relative PO1 fluorescence in MV4-11 cells treated with U0126 for 16 h at indicated concentrations. **(ii)** Bar chart shows relative mean PO1 fluorescence of treated cells expressed as % of control. Results are representative of four independent experiments. Asterisks indicate statistically significant differences (^*^p<0.05, ^***^p<0.001, ^****^ p<0.0001) as analysed by Student's t-test. Error bars are representative of SD.

The ERK1/2 pathway is known to be activated downstream of constitutively activated FLT3-ITD. We investigated the effect of ERK1/2 signalling inhibition using U0126 in MV4-11 cells (Figure 5A) on NOX4D 28 kDa protein expression. Inhibition of ERK1/2 signalling did not cause a decrease in NOX4 67 kDa and NOX4D 28 kDa protein expression when compared to control, however, p22^phox^ protein expression decreased following treatment with 50 μM and 100 μM U0126 (Figure 5B). This suggests that NOX4 67 kDa- and NOX4D 28 kDa-generated pro-survival ROS are independent of ERK1/2 signalling. A decrease of 40-45% in total endogenous H_2_O_2_ was observed following treatment with 50 μM and 100 μM U0126 (Figure 5C).

### NOX4 67 kDa and NOX4D 28 kDa generate pro-survival ROS downstream of STAT5 signalling

**Figure 6:**
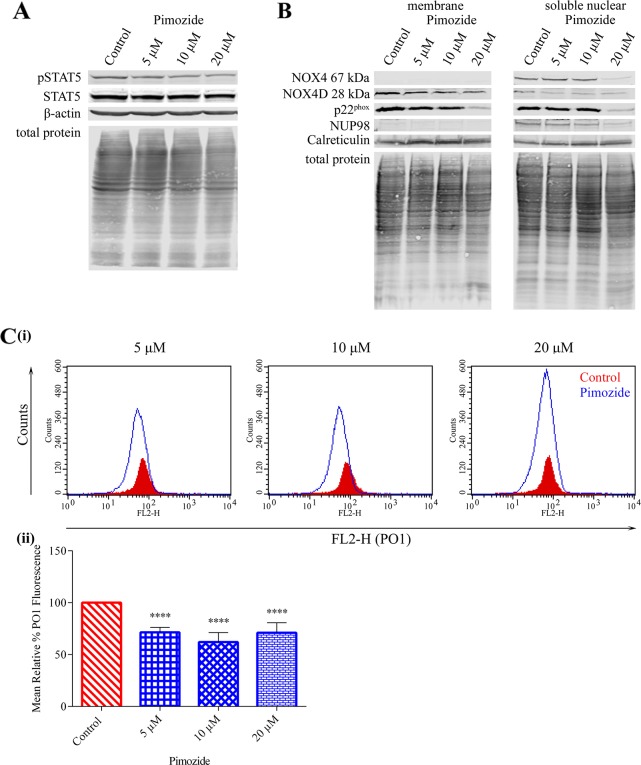
NOX4 67 kDa and NOX4D 28 kDa generate H_2_O_2_ downstream of STAT5 activation **(A)**Western blot analysis of STAT5 signalling in FLT3-ITD expressing MV4-11 cells following treatment with pimozide (5 μM, 10 μM and 20 μM) for 16 h. β-actin and total protein were used as loading controls. **(B)** NOX4 67 kDa, NOX4D 28 kDa and p22^phox^ protein expression in membrane and soluble nuclear fractions of MV4-11 cells following treatment with pimozide for 16 h at indicated concentrations. Equal loading of samples is shown by total protein and verification of subcellular fractions were assessed by probing for nuclear-localised NUP98 and membrane-localised calreticulin. Western blot analysis is representative of four independent experiments. **(C)(i)** Flow cytometric analysis of mean relative PO1 fluorescence in MV4-11 cells treated with pimozide for 16 h at indicated concentrations. **(ii)** Bar chart shows relative mean PO1 fluorescence of treated cells expressed as % of control. Results are representative of three independent experiments. Asterisks indicate statistically significant differences (^****^p<0.0001) as analysed by Student's t-test. Error bars are representative of SD.

Previous studies have shown that FLT3-ITD drives evident activation of STAT5 signalling compared to FLT3-WT resulting in increased mRNA and protein expression of NOX4 [27, 47, 48]. Given that treatment with pimozide clearly reduced NOX4 mRNA in FLT3-ITD expressing MV4-11 cells [27], we investigated the effect of STAT5 inhibition in MV4-11 cells (Figure 6A) on NOX4D 28 kDa protein expression. Treatment with indicated concentrations of pimozide resulted in a pronounced decrease in NOX4 67 kDa, NOX4D 28 kDa and p22^phox^ protein expression following treatment with 20 μM pimozide (Figure 6B). Furthermore, inhibition of STAT5 signalling caused a decrease of 30-40% in total endogenous H_2_O_2_ at all concentrations (Figure 6C), indicating a role for STAT5 signalling in the production of NOX4D-generated pro-survival ROS.

### Inhibition of GSK3β signalling in MV4-11 cell line increases NOX4 67 kDa and NOX4D 28 kDa protein levels and decreases p22^phox^ protein levels

**Figure 7:**
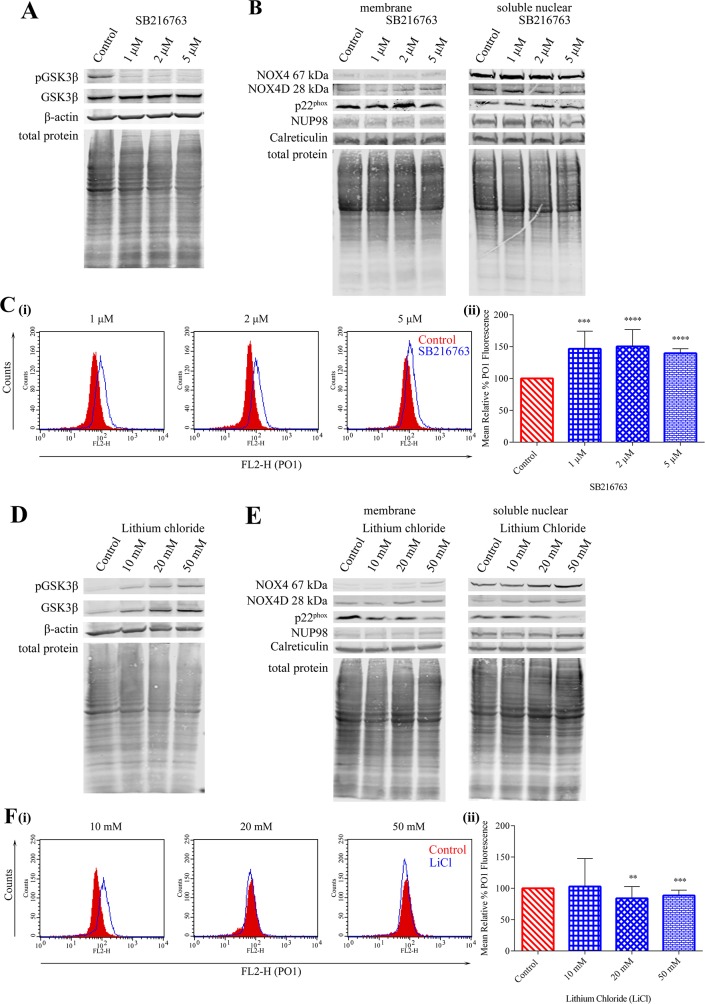
Inhibition of GSK3β signalling results in elevated NOX4 67 kDa and NOX4D 28 kDa protein expression **(A)** Western blot analysis of GSK3β signalling in FLT3-ITD expressing MV4-11 cells following treatment with SB216763 (1 μM, 2 μM and 5 μM) for 16 h. β-actin and total protein were used as loading controls. **(B)** NOX4 67 kDa, NOX4D 28 kDa and p22^phox^ protein expression in membrane and soluble nuclear fractions of MV4-11 cells following treatment with SB216763 for 16 h at indicated concentrations. Equal loading of samples is shown by total protein and verification of subcellular fractions were assessed by probing for nuclear-localised NUP98 and membrane-localised calreticulin. Western blot analysis is representative of four independent experiments. **(C)(i)** Flow cytometric analysis of mean relative PO1 fluorescence in MV4-11 cells treated with SB216763 for 16 h at indicated concentrations. **(ii)** Bar chart shows relative mean PO1 fluorescence of treated cells expressed as % of control. Results are representative of three independent experiments. Asterisks indicate statistically significant differences (^***^p<0.001, ^****^p<0.0001) as analysed by Student's t-test. Error bars are representative of SD. **(D)** Western blot analysis of GSK3β signalling in FLT3-ITD expressing MV4-11 cells following treatment with lithium chloride (10 mM, 20 mM and 50 mM) for 16 h. β-actin and total protein were used as loading controls. **(E)** NOX4 67 kDa, NOX4D 28 kDa and p22^phox^ protein expression in membrane and soluble nuclear fractions of MV4-11 cells following treatment with lithium chloride for 16 h at indicated concentrations. Equal loading of samples is shown by total protein and verification of subcellular fractions were assessed by probing for nuclear-localised NUP98 and membrane-localised calreticulin. Western blot analysis is representative of three independent experiments. **(F)(i)** Flow cytometric analysis of mean relative PO1 fluorescence in MV4-11 cells treated with lithium chloride for 16 h at indicated concentrations. **(ii)** Bar chart shows relative mean PO1 fluorescence of treated cells expressed as % of control. Results are representative of four independent experiments. Asterisks indicate statistically significant differences (^**^p<0.01, ^***^p<0.001) as analysed by Student's t-test. Error bars are representative of SD.

Previous studies in our laboratory have demonstrated that PKC412-mediated p22^phox^ down-regulation requires GSK3β activation, establishing a role for GSK3β signalling in post-translational regulation of NOX4 partner protein p22^phox^ [22]. SB216763, a drug described as an inhibitor of GSK3β [49, 50] was found to decrease pGSK3β (Ser 9) protein levels significantly in MV4-11 cells, therefore resulting in the activation of GSK3β signalling (Figure 7A). Treatment of MV4-11 cells with indicated concentrations of SB216763 showed no obvious effect on NOX4 67 kDa, NOX4D 28 kDa and p22^phox^ protein levels in membrane and soluble nuclear fractions (Figure 7B). However, activation of GSK3β signalling increased total endogenous H_2_O_2_ by 45-50% following treatment with 1 μM and 2 μM SB216763 and ∼40% following treatment with 5 μM SB216763 (Figure 7C). Lithium chloride (LiCl) is widely used as a GSK3β inhibitor [51]. Inhibition of GSK3β using LiCl caused an increase in pGSK3β (Ser 9) protein levels (Figure 7D) in MV4-11 cells, indicative of inhibition of the pathway. This increase coincided with an increase in NOX4 67 kDa and NOX4D 28 kDa proteins in membrane and soluble nuclear fractions. Interestingly, p22^phox^ protein levels decreased following treatment with 50 mM LiCl in both membrane and soluble nuclear fractions (Figure 7E). Inhibition of GSK3β activation had little or no effect on total endogenous H_2_O_2_ levels (Figure 7F).

## DISCUSSION

FLT3-ITD is the most prevalent mutation in AML accounting for 20-30% of patient cases [5, 7, 8] and has been associated with an aggressive phenotype [11–13]. NOX-derived ROS have been shown to have numerous roles in leukaemia including cell survival, cell proliferation and a differentiation block [52, 53]. Leukaemic oncogenes have been widely documented in the regulation of NOX proteins and their partner protein p22^phox^ [8, 54]. Our group has shown that 32D cells transfected with FLT3-ITD possess higher NOX4 and p22^phox^ levels than their wild type counterpart contributing to genomic instability in FLT3-ITD expressing AML. Furthermore, 32D/FLT3-ITD cells exhibit higher levels of total endogenous and nuclear H_2_O_2_ and DNA damage than 32D/FLT3-WT cells [23].

Unlike other NOX family members, NOX4 is constitutively activated. Subcellular localisation of NOX4 is key to its role in ROS production and genetic instability. NOX4 has been reported to be expressed in the nucleus [55–62] amongst other previously identified locations including the cytoskeleton [35], ER [30, 36–38], mitochondria [39, 40] and plasma membrane [38, 41]. Our laboratory has previously shown that NOX4 and p22^phox^ co-localise to the nuclear membrane of FLT3-ITD expressing MV4-11 cells [23]. Previous studies have identified the presence of NOX4 isoforms or splice variants [45]. NOX4D 28 kDa is of particular interest, as it is found in the nucleus and nucleolus of multiple cell types including human aortic smooth muscle cells, HUVECs, H9C2 rat cardiomyocyte cells, HEK cells, mouse primary cardiac fibroblasts and rat neonatal cardiomyocytes where it contributes to ROS production and DNA damage [42].

In this study, we investigated the expression, localisation and regulation of NOX4D-generated pro-survival ROS in FLT3-ITD expressing AML. We found that FLT3-ITD expressing AML patient samples and cells possess the NOX4D splice variant. FLT3-ITD expressing AML cells express NOX4D in the membrane and soluble nuclear fractions. NOX4D was not detected in the FLT3-WT expressing patient samples and cells, suggesting a role for nuclear membrane-localised NOX4D 28 kDa in the generation of pro-survival ROS in FLT3-ITD expressing AML. In line with previous work, we detected NOX4 67 kDa in the soluble nuclear fraction and its partner protein p22^phox^ in the membrane and soluble nuclear fractions of MV4-11 and 32D/FLT3-ITD cells (Figure 1). 32D cells stably transfected with FLT3-ITD express NOX4D 28 kDa and possess elevated levels of total endogenous H_2_O_2_ compared to their wild-type counterpart. Importantly, to our knowledge, this is the first study to identify the role of nuclear membrane-localised NOX4D 28 kDa in pro-survival ROS production and genomic instability in FLT3-ITD expressing AML cells (Figure 1).

p22^phox^ is a partner protein of NOX1-4 and is required for NOX4 activation [30]. We found that p22^phox^ knockdown had no significant effect on NOX4 67 kDa and NOX4D 28 kDa protein levels (Figure 2). Interestingly, previous studies have shown that NOX4 knockdown resulted in depletion of p22^phox^ protein levels in HUVECs with no change in p22^phox^ mRNA expression [43]. This suggests that the formation of NOX4 and p22^phox^ complex in the nucleus of HUVECs is responsible for the stabilisation of p22^phox^ at the protein level.

Recent studies in our laboratory have revealed that NOX4 67 kDa is glycosylated in FLT3-ITD expressing MV4-11 cells [28]. We show that NOX4D 28 kDa is also glycosylated in MV4-11 cells, by using a glycosylation inhibitor, tunicamycin, and a receptor trafficking inhibitor, brefeldin A. By inhibiting glycosylation, NOX4D 28 kDa was observed at a lower molecular weight (Figure 2). Indeed, NOX4D 28 kDa is glycosylated in A549 cells [45]. We have shown that deglycosylation of NOX4 67 kDa [28] and now NOX4D 28 kDa (Figure 2) coincides with significant decreases in total endogenous H_2_O_2_ [28]. This suggests that the glycosylation of NOX4 67 kDa and NOX4D 28 kDa may be important for their role in the production of pro-survival ROS and DNA damage in AML. Tunicamycin and brefeldin A have recently been shown to inhibit receptor trafficking of the FLT3-ITD receptor to the plasma membrane which caused inactivation of both the AKT and ERK1/2 pathways [16, 28]. Although tunicamycin and brefeldin A inhibit many glycosylated proteins, mild inhibition of glycosylation using tunicamycin in combination with FLT3 kinase inhibitors has shown therapeutic potential for the treatment of FLT3-ITD expressing AML [63].

We have previously demonstrated that FLT3-ITD is involved in the up-regulation of NOX4 both at mRNA and protein levels [23, 27]. Inhibition of FLT3-ITD activity using several FLT3 receptor inhibitors including AC220, AG1295 and PKC412 caused a decrease in NOX4 mRNA and protein expression [27, 28] presenting a role for FLT3-ITD in NOX4-generated ROS production in AML. Here, we have shown that FLT3-ITD patient samples and cells express the NOX4D 28 kDa splice variant alongside a dramatic increase in total endogenous H_2_O_2_ compared to their wild-type counterpart that do not express NOX4D (Figure 1). Inhibition of the FLT3 receptor using PKC412 caused a decrease in total endogenous H_2_O_2_ in 32D/FLT3-ITD cells compared to 32D/FLT3-WT cells (Figure 3). FLT3-ITD inhibition in MV4-11 cells treated with PKC412 resulted in down-regulation of NOX4 67 kDa and NOX4D 28 kDa protein expression alongside a decrease in H_2_O_2_ levels (Figure 3). Treatment of MV4-11 and 32D/FLT3-ITD cells with FLT3 receptor inhibitor AC220 also led to decreased expression of NOX4D 28 kDa. PKC412 (midostaurin) has recently been approved by the FDA and AC220 is currently being tested in clinical trials for the treatment of AML [64–66]. Previous studies has shown that FLT3-ITD inhibition using PKC412 and NOX inhibition via diphenyleneiodonium (DPI) in 32D/FLT3-ITD cells resulted in ∼25-40% decrease in γH2AX levels, a marker of dsbs [23]. Together these findings suggest that the FLT3-ITD oncogene is responsible for the regulation and production of nuclear membrane-localised NOX4D-generated H_2_O_2_ in AML contributing to genetic instability and an aggressive phenotype.

Three major pro-survival pathways are activated downstream of FLT3-ITD in AML: PI3K/AKT, ERK1/2 and STAT5 pathway [10, 16]. We have shown that the PI3K/AKT pathway needs to be activated in order for NOX4 to generate its oncogenic effects in AML [27, 28]. Inhibition of the PI3K/AKT pathway revealed that the PI3K/AKT pathway is responsible for the activation and generation of NOX4-, NOX4D- and p22^phox^ generated H_2_O_2_ (Figure 4). Although the ERK1/2 pathway is located downstream of FLT3-ITD, inhibition of ERK1/2 signalling was found to have no noticeable effects on NOX4- and NOX4D- protein expression. However, inactivation of ERK1/2 signalling revealed that ERK1/2 activation is involved in the stimulation and production of p22^phox^-mediated H_2_O_2_ production in AML (Figure 5). p22^phox^ is a partner protein of NOX1, NOX2 and NOX3 as well as NOX4, all of which have a role in ROS production. We have previously demonstrated that NOX2 is also involved in ROS production and DNA damage contributing to genetic instability in FLT3-ITD expressing AML. This same study confirmed that NOX1 does not contribute significantly to ROS production or dsbs [23]. Patient derived FLT3-expressing myeloid cells have been shown to express NOX2, NOX4 and NOX5. However, their murine counterpart have been shown to express NOX1, NOX2 and NOX4. These cells did not express NOX3 [53]. Therefore it is likely that ERK1/2 activation is involved in the production of NOX2-generated H_2_O_2_ in FLT3-ITD expressing AML. Recent findings have identified STAT5 signalling downstream of FLT3-ITD and its requirement for the up-regulation of NOX4-generated H_2_O_2,_ alongside the inactivation of DEP-1 PTP a negative regulator of FLT3 signalling activity [27]. Inhibition of STAT5 signalling revealed that activated STAT5 is required for the production of NOX4-, NOX4D- and p22^phox^-generated ROS (Figure 6). This suggests that NOX4D may also have a role in the partial inactivation of DEP-1 PTP resulting in cellular transformation in FLT3-ITD expressing AML.

GSK3β is another pathway known to be located downstream of FLT3-ITD. Inhibition of the PI3K/AKT pathway in MV4-11 cells resulted in increased expression of pGSK3β (Ser 9) suggesting that the GSK3β pathway is not located downstream of the PI3K/AKT pathway [28]. Phosphorylation of GSK3β at Serine 9 decreased following ERK1/2 inhibition suggesting that the GSK3β pathway is located downstream of ERK1/2 (data not shown). Inhibition of FLT3-ITD signalling caused a decrease in phosphorylation of GSK3β (Ser 9), resulting in increased GSK3β activation which has been shown previously to play a crucial role in the post-translational regulation of p22^phox^ [22, 28]. SB216763 is described as an inhibitor of GSK3β, and GSK3β is inhibited when it is phosphorylated. In disagreement with this, SB216763 was found to decrease levels of pGSK3β in MV4-11 cells (Figure 7), suggesting that it is acting as an activator of GSK3β. It is possible that SB216763 could have different effects in other cell types. However, based on our findings, we advise careful consideration and assessment of pGSK3β levels when using this drug. Activation of GSK3β was found to have no noticeable effect on NOX4-, NOX4D- and p22^phox^ protein expression, however an increase in H_2_O_2_ levels was observed (Figure 7). The activation of GSK3β signalling has been found to be pro-apoptotic in several systems and can provoke mitochondrial injury and this may be responsible for the increase in H_2_O_2_ levels [67–69]. Lithium chloride, also described as an inhibitor of GSK3β, resulted in the expected increase in pGSK3β in MV4-11 cells, indicating inhibition (Figure 7). Inhibition of GSK3β signalling using LiCl revealed an increase in NOX4- and NOX4D-protein expression, however, p22^phox^ protein levels decreased. Moreover, inhibition of GSK3β signalling had little or no effect on total endogenous H_2_O_2_ (Figure 7).

**Figure 8:**
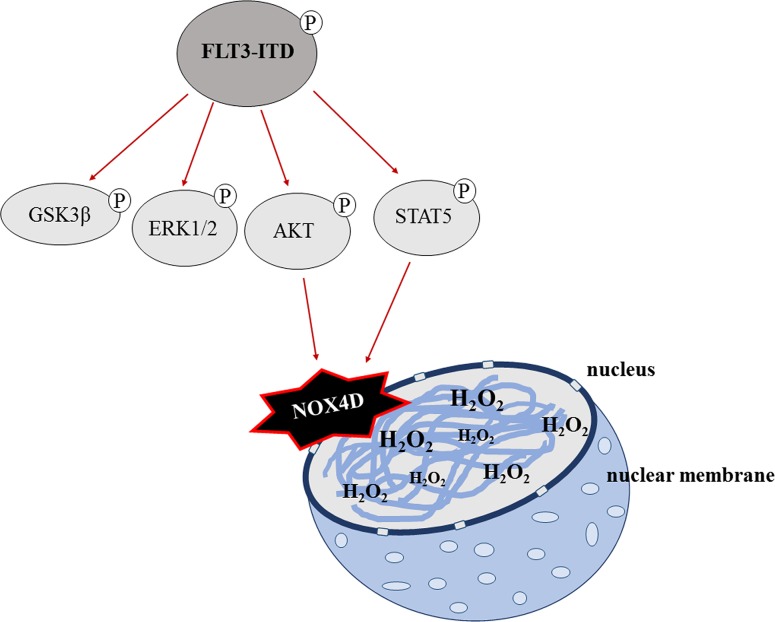
A schematic of the proposed mechanism of FLT3-ITD-driven NOX4D-generated H_2_O_2_ in AML GSK3β, ERK1/2, PI3K/AKT and STAT5 pro-survival pathways are located downstream of FLT3-ITD. Activation of AKT and STAT5 signalling by the FLT3-ITD oncogene results in the activation and production of DNA damaging NOX4D-generated H_2_O_2_ at the nuclear membrane.

In conclusion, we suggest that the FLT3-ITD oncogene is responsible for the activation and generation of NOX4D-generated pro-survival H_2_O_2_ at the nuclear membrane contributing to DNA damage and genetic instability in AML. Glycosylation of NOX4 and NOX4D is essential for the production of pro-survival ROS. Activation of PI3K/AKT and STAT5 signalling is required in order for NOX4D to generate its oncogenic effects. These findings are summarised in Figure 8. This study emphasises the potential of NOX as an effective therapeutic target in FLT3-ITD expressing AML, as inhibition and deglycosylation of NOX4 and NOX4D can decrease the levels of H_2_O_2_ and DNA damage that would otherwise contribute to genetic instability. From a clinical point of view, adaptation of tumour cells to enhanced ROS production may result in tumour cells being less responsive to the standard cytotoxic chemotherapy, which operates through severe oxidative stress. Together the data presented here and in previous studies by our laboratories identifies receptor trafficking inhibitors/glycosylation inhibitors and NOX inhibitors as potential therapies for the treatment of FLT3-ITD expressing AML which when treated in combination with standard chemotherapy may improve the effectiveness of the treatment.

## MATERIALS AND METHODS

### Primary AML patient samples

Blood samples from newly diagnosed untreated *de novo* AML patients were obtained in accordance with the Declaration of Helsinki and with approval of the institutional review board of the University Hospital Jena, Germany. Mononuclear cells were purified as previously described in [21]. FLT3 mutational analysis was done by standard PCR methods to group the patients into FLT3-WT or FLT3-ITD.

### Cell culture and treatments

The human leukaemic cell line MV4-11 (homozygous for the FLT3-ITD mutation) was purchased from DSMZ (Braunschweig, Germany). The 32D cell lines, stably transfected with FLT3-WT and FLT3-ITD [18], were a kind gift from Prof. Hubert Serve (Goethe University Frankfurt, Germany). MV4-11 and 32D cells were maintained in RPMI-1640 medium supplemented with 10% FBS, 2 mM L-glutamine and 1% penicillin/streptomycin in a humidified incubator at 37°C with 5% CO_2_. For the 32D cell lines, 10% WEHI-conditioned medium was added as a source of IL-3. HEK 293-T cells were maintained in Dulbecco's Modified Eagle's Medium (DMEM), supplemented with 10% FBS, 10 mM L-glutamine and 1% penicillin/streptomycin in a humidified incubator at 37°C with 5% CO_2_.

FLT3-ITD was inhibited using PKC412 (50 nM, 200 nM and 250 nM; Tocris) for up to 24 h and AC220 (10nM, 20nM, and 30nM; generous gift of Dr. Siavosh Mahboobi (University of Regensburg, Germany)) for 12 h. Glycosylation was inhibited using tunicamycin (5 μg/ml; Sigma) and brefeldin A (10 μg/ml; Sigma) overnight. Inhibition of PI3K/AKT was achieved via treatment with LY294002 (20 μM, 30 μM and 50 μM; Cell Signaling) for 16 h; inhibition of ERK1/2 via U0126 (10 μM, 20 μM, 50 μM and 100 μM; Sigma) for 16 h; inhibition of STAT5 via pimozide (5 μM, 10 μM and 20 μM; Merck Millipore) for 16 h; modulation of GSK3β via SB216763 (1 μM, 2 μM and 5 μM; Tocris); inhibition of GSK3β via Lithium chloride (LiCl) (10 mM, 20 mM and 50 mM; Sigma) for 16 h. Vehicle controls used were dimethylsulfoxide (DMSO; for all drugs unless otherwise stated), sterile water (for LiCl) and Ethanol (for brefeldin A).

### Antibodies

Primary antibodies used for immunoblotting included NOX4 (Ab109225; Abcam; used for human primary AML samples), NOX4 (NB110-58849; Novus Biologicals; used for all other analyses), p22^phox^ (#SC20781; Santa Cruz Biotechnology), β-Actin (A5441), α-Tubulin (T5168) (Sigma), Vinculin (BZL03106; Biozol), NUP98 (Ab179911 (13C1)), Histone H3 (Ab1220), Calreticulin (Ab22683), KDEL (Ab12223) (Abcam), GAPDH (#5174), Lamin A/C (#2032), HDAC1 (#5356), pAKT (Ser473; #9271), AKT(#9272), pERK1/2 (Thr202/Tyr204; #9106), ERK1/2 (#4696), pGSK3β (Ser9; #9336), GSK3β (#9315) (Cell Signaling), pSTAT5 (Tyr694/699; #04-886; Millipore), STAT5 (#610191; BD Biosciences) and HA (#MMS-101R; Cambridge Bioscience).

### Western blotting

The immunoblotting procedure was carried out as previously described [22]. Briefly, whole cells were lysed in radio-immunoprecipitation assay (RIPA) buffer [Tris–HCl (50mM; pH 7.4), 1% NP-40, 0.25% sodium deoxycholate, NaCl (150mM), EGTA (1mM), sodium orthovanadate (1mM), sodium fluoride (1mM), cocktail protease inhibitors (Roche, Welwyn, Hertforshire, UK) and phenylmethanesulfonyl fluoride (1mM)] for 35-45 minutes at 4°C in a vortex, followed by centrifugation at 14,000 rpm for 15 min to remove cell debris. Subcellular fractionation was performed to detect the expression and localisation of the NOX4 28kDa isoform (NOX4D) using a subcellular fractionation kit (Thermo Scientific, Waltham, MA USA), according to the manufacturer's instructions. The protein concentration was determined using the Bio-Rad Protein Assay (Bio-Rad, Hemel Hempstead, UK). In all cases equivalent amounts of protein, were resolved using SDS-PAGE in 4X Protein Sample Loading Buffer (LI-COR, cat# P/N 928-40004) followed by transfer to nitrocellulose membrane (Schleicher and Schuell, Whatman, Dassel, Germany). Total protein levels were analysed using REVERT total protein stain (LI-COR, cat# P/N 926-11011) as per manufacturer's instructions and imaged on a LI-COR Odyssey infrared imaging system (LI-COR Biosciences UK Ltd, Cambridge, UK). The membranes were blocked for 1 h and incubated overnight with primary antibodies. The membranes were incubated in secondary antibody coupled with Alexa Fluor 680 or 800. Antibody reactive bands were detected using a LI-COR Odyssey infrared imaging system.

### Small interfering RNA (siRNA) and small hairpin RNA (shRNA)

siRNA transfection of MV4-11 cells was performed using the Nucleofector kit L (Amaxa, Cologne, Germany) and Amaxa Nucleofector Technology (Q-001 program) according to the company's protocol. The predesigned siRNA used for p22^phox^ siRNA was A: s201230. The sequences are available from the manufacturer's website. For the negative control, the siRNA used was Silencer Select Negative Control #1 siRNA. All were purchased from Ambion, Warrington, UK. NOX4 siRNA and shRNA in 32D/FLT3-ITD cells were carried out as previously decribed [27].

### Measurement of intracellular H_2_O_2_

Measurement of intracellular H_2_O_2_ was performed as previously described in [28]. Briefly, following treatments, total intracellular H_2_O_2_ was measured by incubating cells with 5 μM of cell-permeable H_2_O_2_-probe PO1 (Tocris) for 1 h at 37°C in the dark. Cells were quantified by flow cytometry using FACSCalibur (BD Biosciences, Europe) and Cellquest Pro software (Becton Dickinson). The mean fluorescent intensity of 10,000 events was determined.

### NOX4 overexpression transfections

For NOX4 overexpression in HEK 293-T cells, cells were transfected using Calcium Phosphate. Briefly, cells were seeded 5 h prior to transfection to allow confluency of 70%. 4 μg of pCMV3-C-HA encoding NOX4 (#HG15189-CY; Sino Biological, UK) was added to CaCl_2_. The DNA/CaCl_2_ mixture was then added dropwise to 2X HBSS at a ratio of 1:1. Samples were allowed to stand for 1-2 min after which the solution was distributed to the pre-seeded cells in a dropwise manner. Cells were then incubated overnight to allow the transfection to proceed, after which cells were reseeded for experimental purposes and lysed 48 h later.

### Statistical analysis

The results are expressed as a percentage of control, set to 100%. Values are representative of mean ± SD and are representative of three or more independent experiments. Statistical significance was analyzed by Student's t-test (GraphPad Prism 6 software) with p<0.05 representing a significant result.

## SUPPLEMENTARY MATERIALS FIGURE



## Abbreviations

AML: acute myeloid leukaemia
chr.b.nuclear: chromatin bound nuclear
DMSO: dimethylsulfoxide
DPI: Diphenyleneiodonium
dsbs: double strand breaks
DUOX: dual oxidase
ER: endoplasmic reticulum
ERK: extracellular signal-regulated kinases
EV: empty vector
FAD: Flavin adenine dinucleotide
FLT3: FMS-like tyrosine kinase 3
FLT3-ITD: FLT3-internal tandem duplication
FLT3-WT: FLT3-wild-type
GSK3β: glycogen synthase kinase-3 β
HEK: human embryonic kidney
HUVEC: human umbilical vein endothelium cells
H_2_O_2_: hydrogen peroxidase
LiCl: Lithium chloride
NOX: nicotinamide adenine dinucleotide phosphate oxidase
p22^phox^: p22 phagocyte oxidase
PI3K: phosphoinositide 3 kinase
PO1: peroxy orange 1
PTP: protein tyrosine phosphatase
RIPA: radio-immunoprecipitation assay
ROS: reactive oxygen species
RTK: receptor tyrosine kinase
STAT5: signal transducer and activator of transcription 5
VSMC: vascular smooth muscle cells
