# Supplementary material for: Nuclear membrane-localised NOX4D generates pro-survival ROS in FLT3-ITD-expressing AML
Source: Oncotarget. 2017 Nov 1;8(62):105440–57. doi: 10.18632/oncotarget.22241 (PMC5739649; doi:10.18632/oncotarget.22241)
Supplement: Supplementary file 1 [file oncotarget-08-105440-s001.pdf]

Nuclear membrane-localised NOX4D generates pro-survival ROS in FLT3-ITD-expressing AML

SUPPLEMENTARY MATERIALS

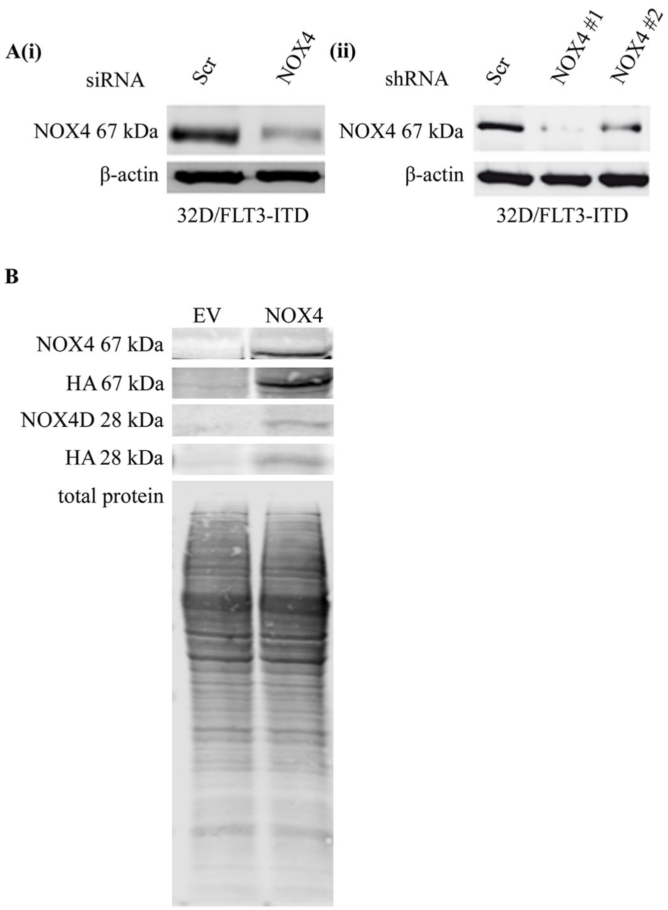

Supplementary Figure 1: NOX4 antibody specificity. Abcam NOX4 antibody (Ab109225) specificity. Western blot analysis of NOX4 67 kDa protein levels in 32D/FLT3-ITD whole cell lysates at 48 h following NOX4 siRNA and shRNA transfection **(A)**. β-actin was used as a loading control. Novus Biologicals NOX4 antibody (NB110-58849) specificity. HEK293-T cells were transfected with EV-HA or pCMV3-C-HA encoding NOX4 using Calcium Phosphate. Western blot analysis of NOX4 67 kDa and NOX4D 28 kDa protein expression in HEK 293-T whole cell lysates following transfection with EV-HA or NOX4-HA for 48 h **(B)**. Total protein was used as a loading control. Blots are representative of three independent experiments.
